# Clinical Study of 30 Novel *KCNQ2* Variants/Deletions in *KCNQ2*-Related Disorders

**DOI:** 10.3389/fnmol.2022.809810

**Published:** 2022-04-26

**Authors:** Tiantian Xiao, Xiang Chen, Yan Xu, Huiyao Chen, Xinran Dong, Lin Yang, Bingbing Wu, Liping Chen, Long Li, Deyi Zhuang, Dongmei Chen, Yuanfeng Zhou, Huijun Wang, Wenhao Zhou

**Affiliations:** ^1^Department of Neonatology, National Children's Medical Center, Children's Hospital of Fudan University, Shanghai, China; ^2^Division of Neurology, National Children's Medical Center, Children's Hospital of Fudan University, Shanghai, China; ^3^Center for Molecular Medicine, National Children's Medical Center, Children's Hospital of Fudan University, Shanghai, China; ^4^Department of Endocrinology and Inherited Metabolic Diseases, National Children's Medical Center, Children's Hospital of Fudan University, Shanghai, China; ^5^Jiangxi Provincial Children's Hospital, Nanchang, China; ^6^Department of Neonatology, People's Hospital of Xinjiang Uygur Autonomous Region, Urumqi, China; ^7^Xiamen Children's Hospital, Xiamen, China; ^8^Quanzhou Women and Children's Hospital, Quanzhou, China

**Keywords:** *KCNQ2*, Kv7.2, newborn, epilepsy, epileptic encephalopathy

## Abstract

**Background:**

*KCNQ2*-related disorder is typically characterized as neonatal onset seizure and epileptic encephalopathy. The relationship between its phenotype and genotype is still elusive. This study aims to provide clinical features, management, and prognosis of patients with novel candidate variants of the *KCNQ2* gene.

**Methods:**

We enrolled patients with novel variants in the *KCNQ2* gene from the China Neonatal Genomes Project between January 2018 and January 2021. All patients underwent next-generation sequencing tests and genetic data were analyzed by an in-house pipeline. The pathogenicity of variants was classified according to the guideline of the American College of Medical Genetics. Each case was evaluated by two geneticists back to back. Patients' information was acquired from clinical records.

**Results:**

A total of 30 unrelated patients with novel variants in the *KCNQ2* gene were identified, including 19 patients with single-nucleotide variants (SNVs) and 11 patients with copy number variants (CNVs). For the 19 SNVs, 12 missense variants and 7 truncating variants were identified. Of them, 36.8% (7/19) of the *KCNQ2* variants were located in C-terminal regions, 15.7% (3/19) in segment S2, and 15.7% (3/19) in segment S4. Among them, 18 of 19 patients experienced seizures in the early neonatal period. However, one patient presented neurodevelopmental delay (NDD) as initial phenotype when he was 2 months old, and he had severe NDD when he was 3 years old. This patient did not present seizure but had abnormal electrographic background activity and brain imaging. Moreover, for the 11 patients with CNVs, 20q13.3 deletions involving *EEF1A2, KCNQ2*, and *CHRNA4* genes were detected. All of them presented neonatal-onset seizures, responded to antiepileptic drugs, and had normal neurological development.

**Conclusion:**

In this study, patients with novel *KCNQ2* variants have variable phenotypes, whereas patients with 20q13.3 deletion involving *EEF1A2, KCNQ2*, and *CHRNA4* genes tend to have normal neurological development.

## Introductions

*KCNQ2* encodes the Kv7.2 subunit of potassium channels. It is located in the neuronal axon initial segment, which plays a critical role in spike initiation (Pan et al., 2006). In the *Kcnq2*-conditional knock-out mice model, the pyramidal neurons located in layer 2/3 (L2/3) were hyperactivated (Niday et al., 2017). Therefore, the *KCNQ2* gene is essential for the regulation of neuronal excitability. In human beings, pathogenic variants in the *KCNQ2* gene could cause benign neonatal seizures and epileptic encephalopathy. Seizure onset usually occurs in the neonatal period. The clinical features of *KCNQ2*-related disorders have a large spectrum of phenotypes, ranging from *KCNQ2*-related benign familial neonatal epilepsy (*KCNQ2*-BFNE) to *KCNQ2*-related neonatal epileptic encephalopathy (*KCNQ2*-NEE) (Numis et al., 2014). Other rare phenotypes, including myokymia, benign familial infantile seizures (BFIS), and infantile spasms, have also been reported in *KCNQ2*-related disorders. The studies reveal that the electroencephalogram (EEG) is characterized as burst-suppression and multifocal epileptic activity (Kato et al., 2013; Lee et al., 2021). Most patients do not present structural abnormality in brain imaging. However, some studies reveal that patients can have thin corpus callosum and abnormal signals in globus pallidus in magnetic resonance imaging (MRI) (Weckhuysen et al., 2012). Regarding management, the response to antiepileptic drugs (AED) also varies (Kuersten et al., 2020). Moreover, the prognostic spectrum is broad in *KCNQ2*-related disorders. Phenotype severity could range from seizure freedom spontaneously to mental developmental delay (Dalen Meurs-van der Schoor et al., 2014). With heterogeneous clinical features, treatment responses, and prognosis, researchers tried to investigate the relationship between the genotype and the phenotype. However, the clear correlation is unknown. In this study, we aim to explore novel candidate variants of *KCNQ2* and provide the related clinical features, AED, and prognosis as well. This information can provide evidence on clinical management in patients with suspected *KCNQ2*-related disorders.

## Methods

### Study Population

In this retrospective study, from January 2018 to January 2021, we enrolled patients with novel pathogenic or likely pathogenic variants of *KCNQ2* or copy number variants (CNVs) covering the *KCNQ2* gene from the China Neonatal Genomes Project (CNGP). All variants were classified according to the guideline of the American College of Medical Genetics (Richards et al., 2015) (Supplementary Table S1). These variants were checked in the Epilepsy Gene project (updated in July 2014; http://www.wzgenomics.cn/EpilepsyGene/), the RIKEE project (updated in December 2015; https://www.rikee.org/), ClinVar (https://www.ncbi.nlm.nih.gov/clinvar/), the Human Gene Mutation Database (HGMD, updated in November 2021, http://www.hgmd.cf.ac.uk).

Clinical data were extracted from medical records, including clinical features, MRI, or EEG findings, and follow-up information in the clinic. The last follow-up was performed by phone call if possible. The study was conducted following the Declaration of Helsinki (as revised in 2013). The Children's Hospital of Fudan University ethics committee approved this study since the study began (No. 2020-227). Pretest counseling was performed by physicians and geneticists. Informed consent was obtained from the patients' parents.

### Next-Generation Sequencing and Sanger Confirmation

Sequences were generated using the Agilent ClearSeq Inherited Disease Kit, Illumina Cluster, and SBS Kit and performed on an Illumina HiSeq 2000/2500 platform. The detected variants were confirmed using polymerase chain reaction (PCR) and PCR-amplified DNA products, which were subjected to direct automated sequencing (3500XL Genetic Analyzer, Applied Biosystems). *De novo* variants were confirmed by parental evaluation *via* Sanger sequencing. We performed HMZDelFinder (Gambin et al., 2017) and CANOES (Backenroth et al., 2014) for the CNV detection. Each case was evaluated by two geneticists back to back. The annotation and filtrations of both SNVs and CNVs have been described in a published work (Dong et al., 2020).

## Results

### Genetic Analysis of Novel Variants of *KCNQ2* Gene

**Figure 1:**
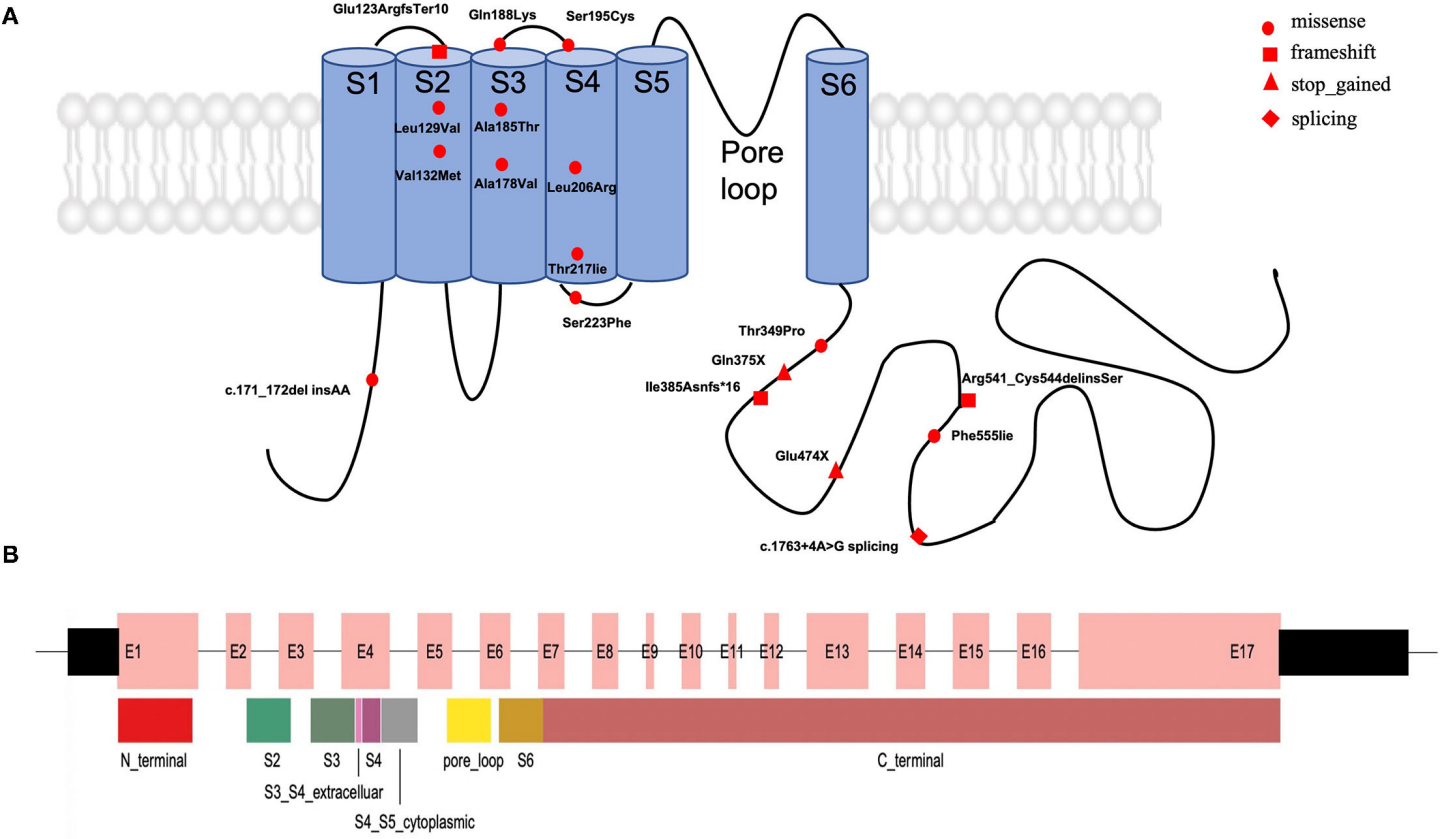
The distribution of the 19 novel variations in the *KCNQ2* gene. **(A)** Approximate locations of the 19 novel *KCNQ2* variants. KCNQ2 protein has six transmembrane domains (blue). The fourth segment acts as the voltage sensor, and the loop between the fifth and sixth domains forms the ion pore in the Kv7.2 potassium channel. **(B)** The distribution of exons and protein domain in the *KCNQ2* gene. The wide box represents the coding region in the 17 exons. S1: segment S1, S2: segment S2, S3: segment S3, S4: segment S4, S5: segment S5, S6: segment S6, S3_S4_extracelluar: the extracellular region between segment S3 and segment S4; S4_S5_cytoplasmic: the cytoplasmic region between segment S4 and segment S5; pore_loop: the loop between the fifth and sixth domains forms the ion pore in a K7.2 potassium channel.

From January 2018 to June 2021, we identified 30 patients with pathogenic variations in the *KCNQ2* gene by the in-house pipeline, including 19 single-nucleotide variants (SNVs) and 11 CNVs. Among the 19 SNVs, one was classified as pathogenic variant, and 18 were likely pathogenic variants (Supplementary Table S1). These variants had not been reported with the detailed clinical phenotypes in the public database. Among them, 12 missense variants, four frameshift variants, two stop-gained variants, and one splicing variant were identified (Figure 1). Nine variants were confirmed as *de novo* variants by Sanger sequencing their parents (Table 1). We identified patient 8 with the variant of c.1623_1631+5del of the *KCNQ2* gene. His father carried a 24% mosaic in his blood, without a seizure history or any neurological phenotype.

**Table 1:** Novel variants in *KCNQ2* gene identified in 19 neonates with *KCNQ2*-related disorders.

**Patient**	**Sex**	**Exon: variant/variation type/domain**	**Inheritance**	**Initial phenotypes/age at seizures onset**	**EEG reports/MRI presentations during neonatal period**	**Treatment received/response to treatment/age at last follow-up/prognosis**
1	F	exon4:c.533C>T: (p.Ala178Val)/ Missense/Segment S3	*De novo*	Generalized tonic-clonic convulsion/3 d	Normal MRI	PB, OXC, LEV, perampanel/ No/9 years/Drug-resistant epilepsy and NDD
2	M	exon3:c.394G>A (p.Val132Met)/Missense/Segment S2	NA	Generalized tonic-clonic convulsion and cyanosis. Several time per day/1 d	NA	Lost follow-up
3	M	exon4:c.617T>G (p.Leu206Arg)/Missense/Segment S4	De novo	Eye deviation to one side and generalized tonic with cyanosis. 1–2 times per day/12 d	Spike-and-slow wave and multifocal spikes with moderate abnormality of EEG background activity/ Abnormal signal in the left basal ganglia, indicating focal leukomalacia and subdural hemorrhage/	PB, TPM, LEV/Yes, seizure-free since 15 MOL/2 years/ Normal ND
4	M	exon4:c.553G>A (p.Ala185Thr)/Missense/Segment S3	De novo	Eye fixation and generalized tonic extension with cyanosis. 3–5 times per day/4 d	Spike-and-slow wave and multifocal spikes with mild abnormality of EEG background activity. Asymmetric/ Normal MRI	PB, LEV/Yes, seizure-free since 8 MOL/8 months/Normal ND
5	M	exon13:c.1420G>T (p.Glu474X)/ Stop_gain/C-terminal region	NA	Eye blinking with generalized clonic components. Several times per day/2 d	NA/Normal MRI	PB/Yes, seizure-free since 12 MOL/3 years/Normal ND
6^*^	M	exon2:c.385C>G (p.Leu129Val)/ Missense/Segment S2	*De novo*	Seizures with cyanosis. 3 times/1 d/	Multifocal spikes in frontal and Rolandic areas with mild abnormality of EEG background activity/ Normal MRI	Lost follow-up
7^*^	M	exon2:c.367delG (p.Glu123ArgfsTer10)/ Frameshift/Segment S2	NA	Seizures since neonatal period. Daily/4 d	Normal EEG background activity/ Normal MRI	Lost follow-up
8^*^	M	exon14:c.1623_1631+5del (p.Arg541_Cys544delinsSer)/Frameshift/C-terminal region	Paternal (mosaic)	Generalized tonic extension with cyanosis and apnea. 3 times before admission/1 d	NA	Lost follow-up
9	M	exon4:c.668C>T (p.Ser223Phe)/Missense/C-terminal region	NA	Preterm infant. Eye fixation and generalized clonic components with cyanosis. 1–2 times per day/9 d	Spike-and-slow wave and multifocal spikes with moderate abnormality of EEG background activity/ Normal MRI	Lost follow-up
10	M	exon1:c.171_172del insAA/ Frameshift/N-terminal region	De novo	Poor head control at 2 months of age, but no seizures movements were observed.	Abnormal EEG background activity/Reduced number of sulci, wide gyri, and delayed myelination	3 years/NDD
11	F	exon4:c.562C>A (p.Gln188Lys)/ Missense/Extracellular	NA	Eye fixation and generalized clonic components with cyanosis/15 d	Spike-and-slow wave and multifocal spikes with severe abnormality of EEG background activity	PB, OXC/Yes, seizure-free since 4 MOL/2.5 years/ Normal ND
12	M	exon8:c.1045A>C (p.Thr349Pro)/Missense/C-terminal region	NA	Generalized clonic components with cyanosis. Severe time per day/3d	Abnormality of EEG background activity/Normal MRI	PB, VitB6/Yes/Lost follow-up
13	M	exon10:c.1154dupA (p.IIe385Asnfs^*^16)/Frameshift/C-terminal region	*De novo*	Eye fixation or deviation to one side, generalized tonic-clonic convulsion with cyanosis. Several times per day/2 d	SB with severe abnormality of EEG background activity	PB, VitB6/ Lost follow-up
14	M	exon15:c.1663T>A (p.Phe555Iie)/Missense/C-terminal region	*De novo*	Seizures since neonatal period. Daily/3d	Multifocal spikes with an abnormality of EEG background activity/Normal MRI	PB, LEV, VPA/Yes/ Lost follow-up
15	M	exon9:c.1123C>T (p.Gln375X)/ Stop_gained/C-terminal region	NA	Seizures since neonatal period. More than 10 times per day/4d	Multifocal spikes with abnormality of EEG background activity/ Normal MRI	VPA/ Yes/ 1 year/ Normal ND
16^*^	M	exon15:c.1763+4A>G/ splicing/C-terminal region	NA	Generalized tonic-clonic convulsion with cyanosis. 4 times before admission/8 h	Normal EEG background activity/ Normal MRI	PB, TPM/Yes/Lost follow-up
17	F	exon4:c.650C>T (p.Thr217Iie)/ Missense/Segment S4	*De novo*	Eye deviation to one side, generalized clonic components with bradycardia and cyanosis/10 d	Normal EEG background/ Delayed myelination and subarachnoid hemorrhage	PB/Yes/Lost follow-up
18	M	exon4: c.584C>G (p.Ser195Cys)/ Missense/Extracellular	*De novo*	Cyanosis with generalized tonic extension. Several times per day/2 d	Spike-and-slow wave and multifocal spikes with moderate abnormality of EEG background activity	PB, VitB6/presented seizure with PB and vitB6 during hospitalization and lost follow-up.
19	M	exon4:c.650C>T (p.Thr217Iie)/ Missense/Segment S4	NA	Generalized tonic extension/1 d	NA	Lost follow-up

*MOL, months of life; ND, neurological development; NDD, neurodevelopmental delay; PB, phenobarbital; TPM, topiramate; LEV, Levetiracetam; VPA, valproic acid; OXC, Oxcarbazepine*.

*SB, suppression-burst pattern; CPAP, Continuous Positive Airway Pressure; NA, not available*.

* *These neonates had a family history. Patient 6: his mother had seizures in childhood. Patient 7: his father was diagnosed with schizophrenia and his mother had an intellectual disability. Patient 8: his sister was diagnosed with epilepsy managed by an antiepileptic drug. She has been seizure-free since 4 months old. Patient 16: his brother and mother were diagnosed with epilepsy. His brother was managed by antiepileptic drug and seizure-free since one-year-old. His mother's aunt was diagnosed with epilepsy and her son and daughter also*.

For the 19 variants, 36.8% (7/19) of the *KCNQ2* variants were located in C-terminal regions, 15.7% (3/19) in segment S2, and 15.7% (3/19) in segment S4. The variant of c.171_172delinsAA located in the N-terminal region, two variants (exon4:c.562C>A and exon4:c.584C>G) in the extracellular region, three variants (exon3:c.394G>A, exon2:c.385C>G, and exon2:c.367delG) in segment S2, two variants (exon4:c. 533C>T and exon4:c.553G>A) in segment S3, three variants (exon4:c.617T>G, exon4:c.650C>T; and exon4:c.650C>T) in segment S4, one variant (exon4:c.668C>T) in cytoplasmic domain between segment S4 and segment S5, and seven variants (exon13:c.1420G>T, exon14:c.1623_1631+5del, exon8:c.1045A>C, exon10c.1154dupA, exon15:c.1663T>A, exon9:c.1123C>T, and exon15:c.1763+4A>G) in C-terminal region (Figure 1).

We also detected 11 patients with 20q13.3 deletion. The size of deletion ranges from 59 kb to 1.8 Mb. In this region, three genes including *KCNQ2, EEF1A2*, and *CHRNA4* were related to dominant epileptic encephalopathy, and *KCNQ2* is the key gene. Seven patients had a continuous deletion of *EEF1A2, KCNQ2*, and *CHRNA4* genes; three had a deletion of *CHRNA4* and *KCNQ2* genes; one had a deletion of *KCNQ2* and *EEF1A2* genes.

### Clinical Features of Patients With *KCNQ2* Variants

Seizures are the dominant and initial features (29/30, 96.7%) in this cohort (Tables 1, 2). The onset time of seizures ranged from 8 h of life to 15 days of life. The most common EEG finding is spike-and-slow wave and multifocal spikes with mild-to-severe abnormality of EEG background activity. Eight patients had positive MRI findings, showing abnormal signal in the left basal ganglia (patient 3), hypoplasia of the brain (patient 10), delayed myelination (patient 17), left ventricle enlargement (patient 20 and patient 21), an abnormal signal in the right frontal lobe (patient 24), and dilation of bilateral ventricles (patient 27 and patient 28). All patients with 20q13.3 deletion presented tonic seizures or tonic-clonic seizures during the neonatal period. Moreover, there were no significant different motor manifestations or imaging findings between the groups with SNVs and CNVs in the neonatal period.

**Table 2:** Novel deletion in *KCNQ2* gene identified in 11 neonates with *KCNQ2*-related disorders.

**Patient**	**Sex**	**Chromosome: position (start-end); size (covered genes)**	**Initial phenotypes/age at seizures onset**	**EEG reports/MRI presentations during neonatal period**	**Treatment received/response to treatment/age at last follow-up/prognosis**
20^*^	M	Chr20: 61944468-62104030; 159 kb (*CHRNA4, KCNQ2*)	Upper limb tonic extension/3 d	Moderate abnormality of EEG background activity/ Left ventricle enlargement	PB/Yes. Seizure free since 9 MOL/ 16 m/Normal ND
21	F	Chr20: 62069977-62129187; 59 kb (*KCNQ2, EEF1A2*)	Apnea/2 d	NA/ Left ventricle enlargement	PB/Yes. No seizure after discharging home/2 y/Normal ND
22^*^	M	Chr20: 61974574-62129187; 154 kb (*CHRNA4, KCNQ2, EEF1A2*)	Upper limb tonic extension with lower limb tonic extension. Three times before admission/2 d	Normal EEG/ Normal MRI	PB/Yes. No seizure after discharging home/2 y/Normal ND
23	M	Chr20:61974574-62129187; 154 kb (*CHRNA4, KCNQ2, EEF1A2*)	Apnea assisted by CPAP, followed by generalized tonic extension. Several times per day/3 d	Spike-and-slow wave and multifocal spikes with mild abnormality of EEG background activity/ Normal MRI	PB, LEV/No/5 m/Drug-resistant epilepsy but Normal ND
24	M	Chr20: 61974574-62078190; 103 kb (*CHRNA4, KCNQ2, EEF1A2*)	Eye deviation to one side, generalized clonic components with cyanosis and tachycardia. Daily/2 d	Spike-and-slow wave and multifocal spikes with moderate abnormality of EEG background activity/ Subdural hemorrhage, and focal injury of the right frontal lobe	PB/Yes. Seizure-free since 3 MOL/ 14 m/Normal ND
25^*^	F	Chr20: 61986847-62055559; 68 kb (*CHRNA4, KCNQ2*)	Eye fixation and generalized clonic components/3 d	Spike-and-slow wave and multifocal spikes with abnormality of EEG background activity	Lost follow-up
26	F	Chr20: 61041481-62680992; 1.6 Mb (*EEF1A2, KCNQ2, CHRNA4*)	Generalized clonic components/2 d	NA/NA	PB/Yes. Lost follow-up
27	F	Chr20:61273854-62907579; 1.6 Mb (*EEF1A2, KCNQ2, CHRNA4*)	Eye fixation and generalized tonic extension with bradycardia and cyanosis. Daily/2 d	Spike-and-slow wave and multifocal spikes with severe abnormality of EEG background activity. Asymmetric/ Dilation of the bilateral ventricles	PB, LEV/Yes. Seizure-free since 3 MOL/ 4m/Normal ND
28	F	Chr20: 61038552-62907579; 1.8Mb (*EEF1A2, KCNQ2, CHRNA4*)	Eye deviation to one side, generalized tonic extension with cyanosis. Several times before admission/3 d	Moderate abnormality of EEG background activity/ Dilation of bilateral ventricles, multiple ependymomas in the bilateral ventricles	PB, OXC/Yes. Seizure-free since 9 MOL/ 9 m/Normal ND
29^*^	F	Chr20: 61986847-62224435; 68kb (*CHRNA4, KCNQ2*)	Generalized tonic extension with cyanosis. 3-4 times per day/8d	Normal EEG/ Normal MRI	PB/Yes. No seizure after discharging home/10 m/Normal ND
30^*^	F	Chr20: 61826780-2660844; 834kb (*EEF1A2, KCNQ2, CHRNA4*)	Eye deviation to one side, generalized tonic extension with cyanosis. 3–4 times per day/3 d	Spike-and-slow wave and multifocal spikes with moderate abnormality of EEG background activity	PB, TPM/Yes. Seizure-free since 3 MOL 4 m/Normal ND

*MOL, months of life; ND, neurological development; NDD, neurodevelopmental delay; PB, phenobarbital; TPM, topiramate; LEV, Levetiracetam; VPA, valproic acid; OXC, Oxcarbazepine*.

*SB, suppression-burst pattern; CPAP, Continuous Positive Airway Pressure; NA, not available*.

* *These neonates had a family history. Patient 20: both his mother and aunt presented seizures, but were improved without medication. Patient 25: both her father and grandmother presented seizures in their childhood. Patient 29: her brother presented seizures after birth and was resolved. Patient 30: her mother presented seizures in childhood*.

Among them, we found a male term patient (patient 10) presented with motor developmental delay as the initial phenotype when he was 2 months old. He was born uneventfully. He was diagnosed with pneumonia after birth and admitted to the neonatal department. He presented poor head control at 2 months of age, and he was referred to a local children's hospital. He could sit unsupported until 10 months old and was diagnosed with motor developmental delay. Cranial MRI showed a reduced number of sulci, wide gyri, and delayed myelination. The EEG finding is abnormal. As the EEG was performed after the onset of motor developmental delay disorder, whether the EEG was positive at the early stage was not available. The seizure and tremor phenotype of this patient is negative (information from his mother).

### Clinical Management and Prognosis of Patients With *KCNQ2* Variants

The overall prognosis was favorable for the patients with follow-up in the clinic. Among the 19 patients with SNVs, nine patients were responsive to AED and seizure-free by 2 years old, one patient (patient 1, segment S3, p.Ala178Val) had drug-resistant epilepsy, one patient (patient 10) did not present seizure, and eight patients lost follow-up. Among the 9 patients who were seizure-free, the regions where the variants were located included two segment S4 regions (patient 3, p.Leu206Arg; patient 17, p.Thr217Iie), 1 segment S3 (patient 4, p.Ala185Thr), one extracellular region (patient 11, p.Gln188Lys), and 5 C-terminal regions (patient 5, p.Glu474X; patient 12, p.Thr349Pro; patient 14, p.Phe555Iie; patient 15, p.Gln375X; and patient 16, exon15:c.1763+4A>G). Regarding the prognosis, two patients (patient 1 and patient 10) presented neurodevelopmental delay (NDD), nine patients had normal neurological development, and eight patients lost follow-up.

Among the 11 patients with 20q13.3 deletion, nine patients were responsive to AED, and eight of them were seizure-free by 2 years old. The remaining two patients, one (patient 23) had drug-resistant epilepsy, and one (patient 25) lost follow-up. Regarding the prognosis, one (patient 25) had NDD. Different from the variable outcomes of patients with SNVs, all patients with 20q13.3 deletions with available information had normal neurological development.

## Discussion

We report 30 unrelated patients with novel variants in the *KCNQ2* gene, including 19 SNVs and 11 CNVs. For SNVs, missense was the most common mutation type (63.2%, 12/19), and 36.8% (7/19) of the *KCNQ2* variants were located in C-terminal regions in our cohort. Mosaic parents in *the KCNQ2* gene were reported in the literature, the mosaic state of asymptomatic parents is from 5% to 28% (Milh et al., 2015). One father with 30% mosaicism had a neurological phenotype (Weckhuysen et al., 2012). This information may indicate that mosaicism could not be ignored in epileptic encephalopathy. Furthermore, parental carrier testing should be considered regarding suffering. The next baby may still have a chance to inherit the pathogenic variant and will be affected.

In our study, all but one patient (patient 10 with the variant of c.171_172delinsAA) presented seizures in the neonatal period. Patient 10 presented motor developmental delay as an initial clinical feature. Ten EEG findings showed multifocal epileptiform with an abnormality of background activity. However, the clear phenotype–genotype correlation is unknown (Malerba et al., 2020). Previous studies indicated that *KCNQ2* missense variants were associated with severe epilepsy phenotype and poor neurological outcomes because of dominant-negative effects (Orhan et al., 2014), whereas truncating variants were likely to be *KCNQ2*-BFNE (Soldovieri et al., 2007). Research suggested that the phenotype of patients was not only related to the mutation type but also associated with the affected regions of *KCNQ2* (Goto et al., 2019). For example, missense variants in segment S6 and its nearby regions are likely to result in poor neurological outcomes (Goto et al., 2019). However, in our study, patient 1 with missense variant located in segment S3 had NDD, but patient 4 also with missense variant located in segment S3 had normal neurological development (aged 8 months old). Other patients with missense variants located in segment S4 also had normal neurological development. Therefore, the characteristics of pathogenic variants are still difficult to be linked to their clinical characteristics.

Consistent with previous studies, the patients who were responsive to AED could have variants located in segment S2 (Soldovieri et al., 2019), pore-loop domain (Weckhuysen et al., 2012; Pisano et al., 2015; Gomis-Perez et al., 2019), segment S4 (Weckhuysen et al., 2012; Pisano et al., 2015), segment S6 (Abidi et al., 2015; Pisano et al., 2015), C-terminal region (Weckhuysen et al., 2012; Pisano et al., 2015; Lee et al., 2017; Gomis-Perez et al., 2019), and extracellular region (Weckhuysen et al., 2012). Moreover, one patient (patient 4) with a variant located in segment S3 was responsive to AED and was seizure-free since he was 8 months old.

Neurodevelopmental delay often onset after seizure in *KCNQ2*-related disorders. In this study, we reported one patient had NDD as the initial phenotype. Then, the EEG was abnormal. No tremor or seizure was observed in this patient. This patient carried a *de novo* frameshift variant (c.171_172delinsAA). This variant was ranked as a likely pathogenic variant (Supplementary Table S1). Apart from NDD, the *KCNQ2* gene is also related to autism (Millichap et al., 2016; Long et al., 2019). This study is reported in patients and was proved by the animal model (Kim et al., 2020). Therefore, the *KCNQ2* gene may also be the candidate gene in patients with social behavior abnormalities in clinical genetic counseling.

The 20q13.3 microdeletion syndrome is characterized as seizure, brain abnormalities, NDD, and psychological problems (Pascual et al., 2013). There are variable phenotypes of 20q13.33 deletion (Kurahashi et al., 2009; Traylor et al., 2010; Mefford et al., 2012). The severe neurological phenotypes include learning disability, hyperlaxity, and strabismus (Béna et al., 2007). In this study, we reported a mild phenotype in 11 patients with 20q13.3 deletion involving *EEF1A2, KCNQ2* and *CHRNA4* genes. These clinical features are similar to BFNE caused by *KCNQ2* variations and are different from those of autosomal-dominant nocturnal frontal lobe epilepsy (ADNFLE) caused by *CHRNA4* variations (Steinlein et al., 1995) and developmental and epileptic encephalopathy 33 (DEE33) caused by *EEF1A2* variations (Carvill et al., 2020). Moreover, the dosage sensitivity curations of the above three genes in the ClinGen (https://search.clinicalgenome.org/kb/gene-dosage?page=1&size=25&search=) suggested that *KCNQ2* gene had sufficient evidence for haploinsufficiency and was ranked as the top 1 causative gene based on gnomAD pLI score and gnomAD predicted loss-of-function, whereas the other two genes were not yet evaluated. Therefore, the *KCNQ2* gene is considered the causative gene of the patients with 20q13.3 deletions in our study.

Consistent with a previous study (Okumura et al., 2015), 20q13.3 deletions are restricted to just *KCNQ2* and *CHRNA4* genes are likely to result in *KCNQ2*-BFNE, and one case with 20q13.3 deletion involving *EEF1A2, KCNQ2*, and *CHRNA4* had normal psychomotor development (Okumura et al., 2015). However, the studies indicated that patients with NDD had a larger deletion of the *KCNQ2* gene (Kurahashi et al., 2009; Traylor et al., 2010; Mefford et al., 2012; Pascual et al., 2013; Okumura et al., 2015). Patient 27 and patient 28 had a large deletion (>1 Mb). They were seizure-free and had normal neurological development. However, both of them were <1 year old at the last visit. Therefore, long-term follow-up will be necessary to determine precise phenotypes. These patients had a milder phenotype than some patients with one single-nucleotide *KCNQ2* pathogenic variant. The underlying reason is elusive and needs to be investigated.

Our study has limitations. The follow-up information was absent in some patients because patients did not present for follow-up in the clinic consistently. Therefore, we cannot diagnose the *KCNQ2*-BFNE or *KCNQ2*-NEE in some patients according to the current information. The EEG and MRI findings were not available because some patients were enrolled from other hospitals, and they could not perform EEG or MRI. Third, our study ended in January 2021. Some patients were <1 year old. Therefore, it will be essential to follow these families up to assess neurological development.

## Conclusion

In conclusion, we reported 30 unrelated patients with novel variations in the *KCNQ2* gene, including SNVs and CNVs. The clinical features and prognosis are heterogeneous in patients with SNVs. However, patients with 20q13.3 deletions restricted to *KCNQ2, CHRNA4*, and *EEF1A2* genes have similar to the phenotypes of BFNE. These findings could assist clinicians in diagnosing and predicting the prognosis of *KCNQ2*-related disorders.

## Data Availability Statement

The datasets presented in this study can be found in online repositories. The names of the repository/repositories and accession number(s) can be found in the article/Supplementary Material.

## Ethics Statement

The studies involving human participants were reviewed and approved by Children's Hospital of Fudan University. Written informed consent to participate in this study was provided by the participants' legal guardian/next of kin. Written informed consent was obtained from the individual(s), and minor(s)' legal guardian/next of kin, for the publication of any potentially identifiable images or data included in this article.

## Author Contributions

TX, XC, YZ, HW, and WZ: conception and design. HW and WZ: administrative support. TX, XC, YX, LY, BW, LC, LL, DZ, and DC: provision of study materials or patients. TX, XC, HC, XD, and HW: collection and assembly of data. XC, XD, and LY: data analysis and interpretation. All authors: manuscript writing and final approval of manuscript.

## Funding

The work was funded by the Clinical Research Plan of SHDC (No. SHDC2020CR4085).

## Conflict of Interest

The authors declare that the research was conducted in the absence of any commercial or financial relationships that could be construed as a potential conflict of interest.

## Publisher's Note

All claims expressed in this article are solely those of the authors and do not necessarily represent those of their affiliated organizations, or those of the publisher, the editors and the reviewers. Any product that may be evaluated in this article, or claim that may be made by its manufacturer, is not guaranteed or endorsed by the publisher.
