# Supplementary material for: Clinical Study of 30 Novel KCNQ2 Variants/Deletions in KCNQ2-Related Disorders
Source: Front Mol Neurosci. 2022 Apr 26;15:809810. doi: 10.3389/fnmol.2022.809810 (PMC9088225; doi:10.3389/fnmol.2022.809810)
Supplement: Supplementary file 1 [file Table_1.DOCX]

**Table S1. The detailed information of the variants detected in our cohort**

| Patient No. | Variations/  Variation type/ | Population frequency based on gnomAD | SIFT_score | Polyphen2_score | MutationTaster_score | ACMG guideline score |
| --- | --- | --- | --- | --- | --- | --- |
| 1 | exon4:c.533C>T:(p.Ala178Val)  missense | 0 | 0.01 | 0.993 | 1 | LP (PM1+PM2+PM6+PP3) |
| 2 | exon3:c.394G>A(p.Val132Met)  Missense | 0 | 0.009 | 0.913 | 1 | LP (PM1+PM2+PP3) |
| 3 | exon4:c.617T>G(p.Leu206Arg)  missense | 0 | 0 | 0.999 | 1 | LP (PM1+PM2+PM6+PP3) |
| 4 | exon4:c.553G>A(p.Ala185Thr)  missense | 0 | 0.007 | 0.769 | 1 | LP (PM1+PM2+PM6+PP3) |
| 5 | exon13:c.1420G>T(p.Glu474X)  stop_gained | 0 | / | / | 1 | LP (PVS1+PM2) |
| 6 | exon2:c.385C>G(p.Leu129Val)  missense | 0 | 0.023 | 0.999 | 0.997 | LP (PM1+PM2+PM6+PP3) |
| 7 | exon2:c.367delG(p.Glu123ArgfsTer10)  frameshift | 0 | / | / | / | LP (PVS1+PM2) |
| 8 | exon14:c.1623_1631+5del  (p.Arg541_Cys544delinsSer)  frameshift | 0 | / | / | . | LP (PVS1+PM2) |
| 9 | exon4:c.668C>T(p.Ser223Phe)  missense | 0 | 0 | 0.992 | 1 | LP (PM1+PM2+PM5+PP3) |
| 10 | exon1:c.171_172delinsAA  frameshift | 0 | / | / | / | LP (PS2+PM2) |
| 11 | exon4:c.562C>A(p.Gln188Lys)  missense | 0 | 0.003 | 0.932 | 1 | LP (PM1+PM2+PP3) |
| 12 | exon8:c.1045A>C(p.Thr349Pro)missense | 0 | 0.001 | 0.987 | 1 | LP (PM1+PM2+PM5+PP3) |
| 13 | exon10:c.1154dupA(p.IIe385Asnfs*16)frameshift | 0 | / | / | / | P (PVS1+PM2+PM6) |
| 14 | exon15:c.1663T>A(p.Phe555Iie)missense | 0 | 0 | 0.998 | 1 | LP (PM1+PM2+PM6+PP3) |
| 15 | exon9:c.1123C>T(p.Gln375X)  stop_gained | 0 | / | / | / | LP (PVS1+PM2) |
| 16 | exon15:c.1763+4A>G  splicing | 0 | / | / | / | LP (PM2+PS2) |
| 17 | exon4:c.650C>T(p.Thr217Iie)  missense | 0 | 0 | 0.968 | 1 | LP (PM1+PM2+PM6+PP3) |
| 18 | exon4:c.584C>G(p.Ser195Cys)  missense | 0 | 0 | 0.462 | 1 | LP (PM1+PM2+PM5+PM6+PP3) |
| 19 | exon4:c.650C>T(p.Thr217Iie)  missense | 0 | 0 | 0.968 | 1 | LP (PM1+PM2+PM5+PP3) |

ACMG: American College of Medical Genetics, P: pathogenic variant, LP: likely pathogenic variant.
